# Supercell refinement: a cautionary tale

**DOI:** 10.1107/S2059798319011082

**Published:** 2019-08-28

**Authors:** Jeffrey Lovelace, Václav Petrícek, Garib Murshudov, Gloria E. O. Borgstahl

**Affiliations:** a The Eppley Institute for Research in Cancer and Allied Diseases, 987696 Nebraska Medical Center, Omaha, NE 68198-7696, USA; b Structures and Bonding, Institute of Physics, Academy of Sciences of the Czech Republic, Cukrovarnická 10, 162 53 Praha 6, Czech Republic; c MRC Laboratory of Molecular Biology, Francis Crick Avenue, Cambridge CB2 0QH, England

**Keywords:** aperiodic crystallography, supercell crystallographic refinement, modulated protein crystals, superspace group

## Abstract

Theoretically, crystals with supercells exist at a unique crossroads where they can be considered as either a large unit cell with closely spaced reflections in reciprocal space or a higher dimensional superspace with a modulation that is commensurate with the supercell. In the latter case, the structure would be defined as an average structure with functions representing a modulation to determine the atomic location in 3D space. Here, a model protein structure and simulated diffraction data were used to investigate the possibility of solving a real incommensurately modulated protein crystal using a supercell approximation. In this way, the answer was known and the refinement method could be tested. Firstly, an average structure was solved by using the ‘main’ reflections, which represent the subset of the reflections that belong to the subcell and in general are more intense than the ‘satellite’ reflections. The average structure was then expanded to create a supercell and refined using all of the reflections. Surprisingly, the refined solution did not match the expected solution, even though the statistics were excellent. Interestingly, the corresponding superspace group had multiple 3D daughter supercell space groups as possibilities, and it was one of the alternate daughter space groups that the refinement locked in on. The lessons learned here will be applied to a real incommensurately modulated profilin–actin crystal that has the same superspace group.

## Abstract 2

A higher dimensional superspace description accounts for an unexpected supercell refinement result.

## 1. Introduction   

**Figure 1:**
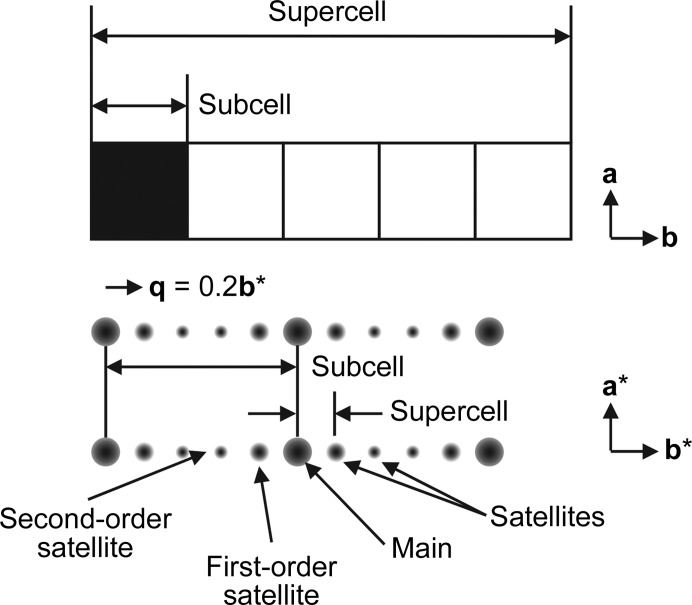
Relationship between the reflections and unit cells. A 5× supercell composed of five subcells in the **b** and b* directions, as well as the corresponding interpretations in diffraction space where there are main reflections and satellite reflections, which could alternatively be assigned as associated with the subcell and supercell. In this example, there are four satellite reflections (two first-order and two second-order reflections) per main reflection along the b* direction.

**Figure 2:**
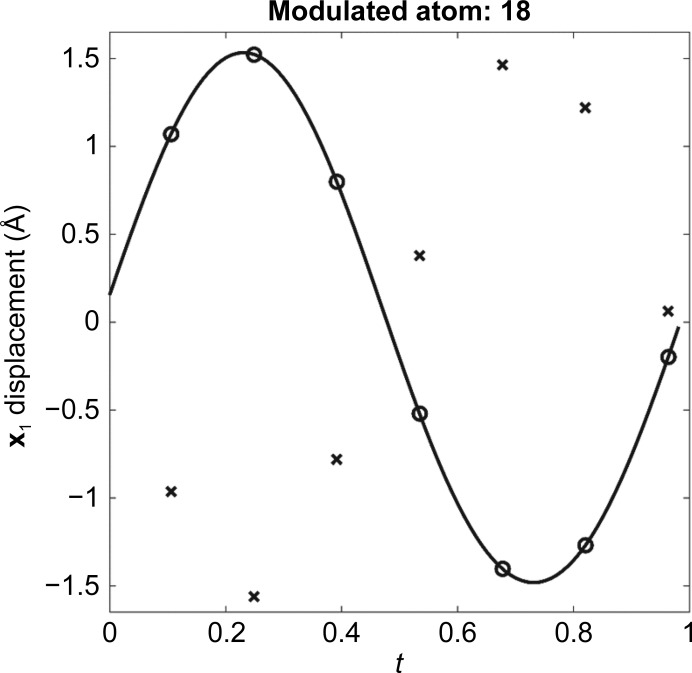
A *t* plot showing the AMF (black line), the correct supercell positions (circles) and the refined supercell positions (crosses).

On occasion, a diffraction pattern is observed that consists of many intense reflections interspersed with many weaker ‘satellite’ reflections. Indexing software may find a good fit for the main (most intense) reflections and may not index the weaker reflections. In some of these cases, this subcell can be extended in integer multiples along one or more of its dimensions, forming a supercell, so that all of the reflections can be correctly indexed (Fig. 1 ▸). When the satellites can be indexed with the main reflections in this manner the diffraction data are called ‘commensurately modulated’. If not indexable, the data are ‘incommensurate’. A view of this process is that the subcell describes an average of what is occurring in the structure if only the main reflections are used, and the other weaker reflections describe the more complex displacement that occurs in each subcell over the supercell. One approach to solving this type of problem is to use the main reflections to arrive at an average solution, and then extend this average solution into a supercell and refine the resulting supercell against all of the reflections. While testing this approach with simulated commensurate data, the expected outcome was that the refined positions, starting from the average position, would match the correct positions that were used to create the reflections for the simulation. After verifying that the approach would work with commensurate data, the plan was then to move on to investigate how commensurate approximates of incommensurate data refine and to see how close the modulation functions match the incommensurate functions (example described below). The refinement had excellent statistics, and initially it was thought to have worked; however, this was not the case. On closer inspection, the expected positions (circles in Fig. 2 ▸) did not match the refined positions (crosses in Fig. 2 ▸). The result appears to be shifted in some way, and this is where the mystery began. Until this issue was resolved, it held up the application of this approach to the ‘real-world’ incommensurate case that we aimed to solve.

### 1.1. Why?   

Why perform the refinement this way? Our research group has been focused on developing approaches to solve modulated protein structures because of an ongoing effort to solve an incommensurately modulated crystal form of profilin–actin (Lovelace *et al.*, 2008 ▸). Progress has been made towards this goal (Porta *et al.*, 2011 ▸, 2017 ▸), but the structure solution has remained problematic. Incommensurate modulation occurs when there is a periodic structural change of some kind overlaid on the crystal lattice, the wavelength of which is not an integer multiple of the unit cell that makes up the crystal. This phenomenon has been described in several of our earlier publications (see Fig. 2 in Lovelace *et al.*, 2013 ▸). A characteristic trait of modulated diffraction is the appearance of weaker satellite reflections around the main reflections. The simplest case is a displacement modulation in which the atomic positions are displaced from the average position by a periodic atomic modulation function (AMF) in superspace. Details of the superspace theory and application of the theory can be found elsewhere (Janner & Janssen, 1977 ▸, 1980 ▸; Janssen *et al.*, 1999 ▸; Smaalen, 2007 ▸). Superspace theory is a very powerful tool. As an example, a single properly chosen superspace group can describe the diversity of crystal forms observed in the solid-matter phase space for a small molecule (Dusek *et al.*, 2003 ▸).

**Figure 3:**
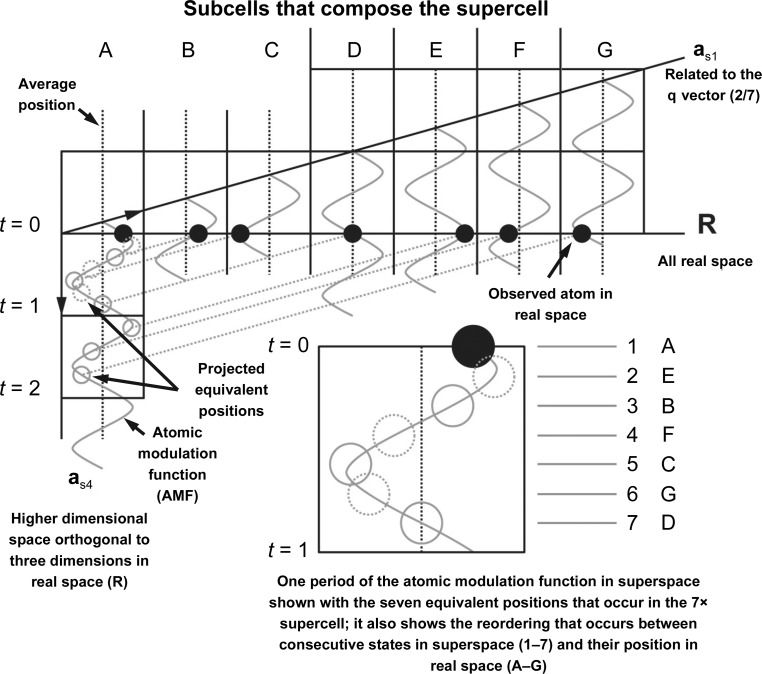
Superspace schematic of a 7× commensurate modulation with two modulation periods every seven subcells. In superspace, when the **q** vector, the periodic AMF and the average position are combined the observed atomic positions in a 7× supercell (subcells A–G) are described. Here, the **q** vector is **q** = (2/7)b*. Through equivalent positions (solid gray circle, projection; dashed gray circle, double projection into *t* = 0–1 cell) all possible states in the crystal can be represented as a single period of the AMF (enlarged area on the lower right). The dimension shown in superspace here is measured in *t* (the phase shift of the AMF) collinear to **a**_s4_, where equal values of *t* run parallel to **R** (an example is the line *t* = 1). Alternatively, the dimension can be measured in fractional **x**_4_ units, collinear to **a**_s4_, where equal values of **x**_4_ run parallel to **a**_s1_ (used to project equivalent positions; the gray lines connecting black filled circles to gray circles).

A schematic diagram of the relationship between superspace and supercells is helpful for understanding the supercell approximation refinement method (Fig. 3 ▸). The atoms (black filled circles) of a supercell appear to be moving randomly in the subcells (A–G). Note that **R** includes all three coordinates of 3D real space (*x*_1_, *x*_2_ and *x*_3_). In higher dimensional space the convention is to represent the directions as **x**_1_, **x**_2_ … **x_*n*_** as opposed to **x**, **y**, **z** or **a**, **b**, **c** because there can potentially be *n* dimensions (many more than are available in normal 3D space). The apparently random motion along **R** can be described as a periodic displacement from an average position (dotted black vertical lines in Fig. 3 ▸) by an AMF in 4D superspace that traverses two periods along the **a**_s4_ direction for every seven subcells. Distances can be represented as *t* fractional units of **x**_4_. There are two common parallel constructions related to **a**_s4_. Lines running parallel to **a**_s1_ have a constant value of *t* and are useful for determining the position that an atom occupies in a unit cell in real space (light gray dotted lines in Fig. 3 ▸). The second projection runs parallel to **R**. These projections are useful for determining atomic distances between pairs of atoms (solid black horizontal lines in Fig. 3 ▸). The AMFs are periodic, and this means that there are multiple equivalent positions. Two ways to translate to equivalent positions are to move to a new **x**_4_ value by transitioning along constructions running parallel to **a**_s1_ (black circles along gray lines to gray circles in Fig. 3 ▸) or to phase shift along **a**_s4_ by moving integer values of *t* (gray circles between *t* = 1 and *t* = 2 to gray dotted circles between *t* = 0 and *t* = 1 in Fig. 3 ▸). Additionally, through the use of equivalent positions and projections, all of the possible positions of an atom in any unit cell in the crystal can be represented within a single period of the AMF (enlarged area in Fig. 3 ▸). Also, it is important to note that states close together in superspace may not be next to each other in real space (1–7 versus A–G in the enlarged portion of Fig. 3 ▸). To avoid further confusion, we also wish to explain that ‘*t*’ is the continouse phase space along **x**_4_, while ‘*T*_0_’ is a shift of where the origin for real space intersects with **x**_4_.

The AMF can be inferred as periodic, as opposed to random, because of the appearance of satellite reflections around the main reflections in the diffraction pattern. For incommensurate cases, normal indexing software can usually index the main reflections but can have difficulty or be unable to index the satellite reflections. In higher dimensional space, satellite reflections are indexed with a **q** vector (Fig. 1 ▸) that describes the direction of the modulation through the crystal as well as its overall frequency (fractional space between the main reflection and its first-order satellite). In special cases, where the modulation becomes commensurate, it is possible to describe the remaining reflections (satellites) by increasing the size of the main or basic unit cell in integer multiples along one or more of the dimensions (Fig. 3 ▸, top). This supercell can then be used with molecular replacement for structure solution, taking care to take translational noncrystallographic symmetry into account (Sliwiak *et al.*, 2014 ▸, 2015 ▸; Campeotto *et al.*, 2018 ▸). For an incommensurate structure, a commensurate approximation (which may also be referred to as a commensurate approximate in the literature) could be used as a way of using the traditional 3D programs to refine the structure by formulating the problem as a supercell. Our hope was that the commensurate approximation to the incommensurate structure would allow us to fit initial AMFs to the atoms and bootstrap the refinement in superspace.

As we had done in the past (Lovelace *et al.*, 2013 ▸), a modulated protein model in a supercell and corresponding 1.0 Å resolution calculated diffraction data were simulated. The standard crystallographic ‘Table 1’ for the simulation was published in Lovelace *et al.* (2013 ▸). These calculations were performed using a combination of *Matlab* (The Mathworks Inc.) and *CCP*4 tools (Winn *et al.*, 2011 ▸). The only portion that used superspace concepts was the calculation of the modulation for each subcell of the supercell. It was desired to make the data behave more like an actual data set in which the model and observed values never perfectly match up, leading to *R* values that were not zero. This was accomplished by trimming the AMFs at second-order Fourier coefficients and trimming the reflections to only include up to second-order satellites. These changes give final *R* values of a few percent instead of zero. In the current work, the simulated diffraction data were important as a first step to study the possibility of using a supercell approximation to solve an incommensurately modulated protein crystal structure and to learn of any potential problems. Owing to software limitations, we were limited to working with commensurate modulations. Simulated data were used so that the focus of the analysis could be on how the refinement approached a known answer as opposed to juggling with other unknowns. We are hopeful that incorporating the results discussed here will lead to a successful pathway to solve the incommensurately modulated profilin–actin complex as well as to improve approaches to refining other macromolecular supercell structures. For those interested in reading further, Wagner & Schönleber (2009 ▸) provide an excellent example of the solution of an incommensurately modulated small molecule using both a commensurate approximation (supercell) and the superspace method to solve the structure.

## 2. Methods   

**Figure 4:**
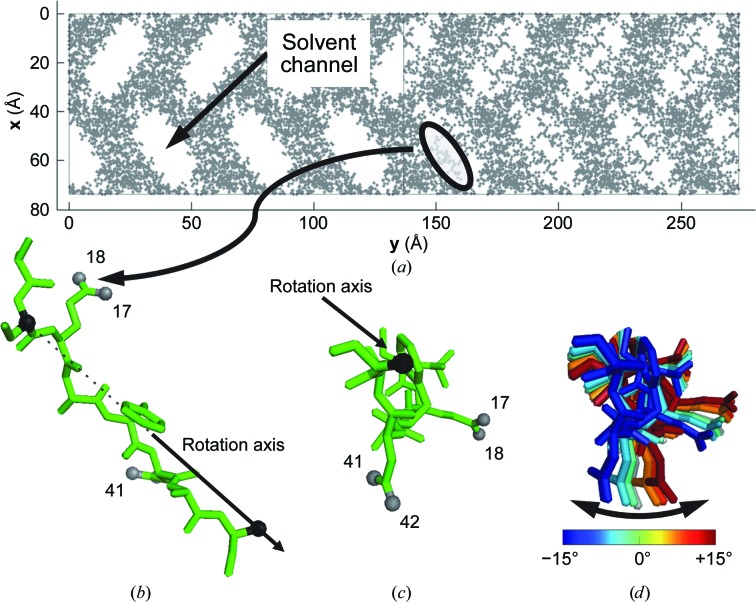
Structural simulation of a modulation. (*a*) A small section of the protein was extracted out into the solvent channel and then modulated as a function of distance in **y** (or **x**_2_). (*b*) The modulation was a simple rotation using two atoms (black spheres) to create a rotation axis, and the gray spheres show the atomic positions that are monitored in Figs. 5 ▸ and 6 ▸. The gray atoms are on parts of the chain with the greatest amount of modulation. (*c*) View looking down the rotation axis. (*d*) The modulation is shown as a rainbow of the seven overlaid modulated chains in the supercell from blue (−15°) to red (+15°). The average position is shown in the stack as a gray molecule located just to the right of the light blue structure and left of the orange structure.

Test structures and simulated diffraction data were made to allow researchers to study refinement strategies for modulated data sets in a controlled setting (Lovelace *et al.*, 2013 ▸). The test data were created from a modified form of the ToxD structure [PDB entry 1dtx; Fig. 4 ▸(*a*)]. The ToxD monomer was broken into three chains. Chain *B* (residues 31–38 of the original molecule), which was located against the solvent channel, was renumbered and translated out into the solvent channel and, to avoid collisions, residues 1, 4, 7 and 8 were mutated to alanines using *Coot* (Emsley *et al.*, 2010 ▸). The coordinates were extended to a 7× supercell, and chain *B* was modulated rotationally around an axis defined by the C^α^ atom in the second residue in chain *B* and the C^β^ atom in the eighth residue in chain *B* [Fig. 4 ▸(*b*)]. The amount of modulation was determined by the location along the **y** direction of the center of mass of chain *B* with a maximum rotation of ±15° [Figs. 4 ▸(*c*) and 4 ▸(*d*)]. The modulating rotation was carried out using *Matlab*. The starting supercell structure for refinement was a 7× expansion of the average structure. The modulation vector for the test diffraction data set was set to **q** = (2/7)b*, or there were two modulation waves every seven unit cells. In other words, each subcell of the supercell had chain *B* in a different rotated orientation based on its position within the supercell. The average structure was found using *Phaser* (McCoy *et al.*, 2007 ▸) to place chains *A*, *B* and *C* into the subcell using only the main reflections. The supercell was refined with *REFMAC* (Murshudov *et al.*, 2011 ▸) using the following settings: restrained refinement for 40 cycles with jelly body enabled and set to 0.020. A zip archive file containing all of the starting models, reflections (mtz) and refined models is available as supporting information and can also be obtained by contacting the corresponding author.

## 3. Results and discussion   

**Figure 5:**
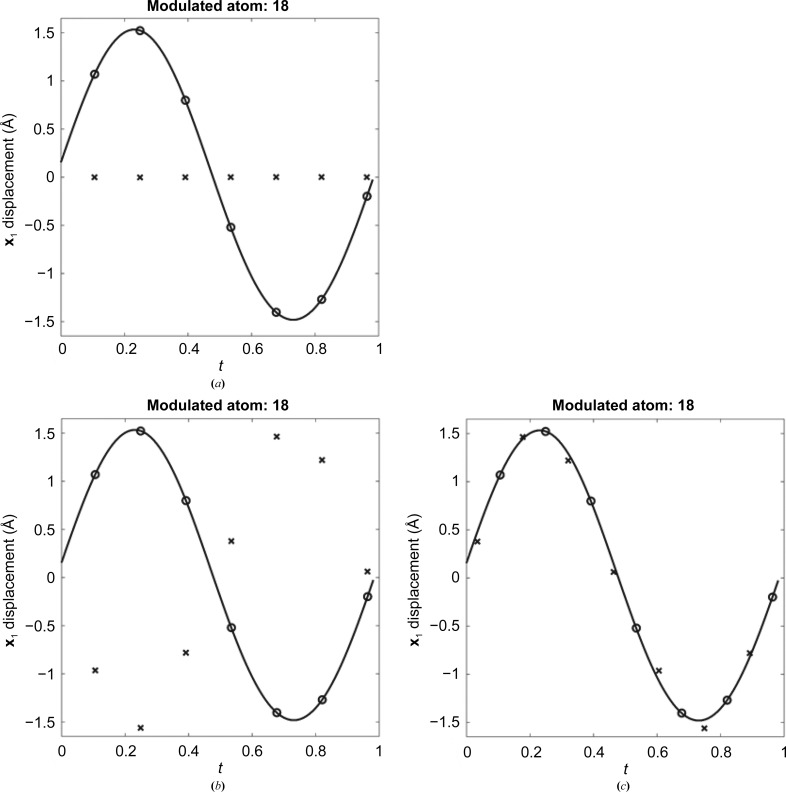
Refinement results. (*a*) The initial state showing the starting position of atom 18 in the **x**_1_ dimension versus *t* (**x**_4_), where *t* from 0 to 1 represents one period of the modulation function. The modulation function is shown as a solid black line, and the expected refined positions are shown as black circles. The starting average positions in the supercell are shown as black crosses, and for an unmodulated atom are on a horizontal line. (*b*) After refinement the black crosses fail to overlay the expected position (black circles) or the AMF (black line). The incorrectly refined positions appear to conform to a sinusoidal-like shape. (*c*) Shifting the incorrectly refined positions to *t* + 1/2 (black crosses) causes them to line up with the known AMF (black line).

The structure solution was performed in two stages. Firstly, the average structure was solved by using only the main reflections and performing molecular replacement with *Phaser* (McCoy *et al.*, 2007 ▸) and was refined with *REFMAC* on the corresponding subcell. The second step was to expand the average solution into a supercell (7× in **y** in this case) and then refine against all reflections that were indexed as a supercell. This approach was taken because it more closely mirrors the formulation of the superspace theory, in which atoms are described mathematically as having an average position that is perturbed by an atomic modulation function, as opposed to directly performing molecular replacement against the entire supercell. When the atoms of a modulated structure in a supercell are plotted as a displacement from their average position as a function of their *t* value in superspace, the resulting seven points (for the supercell used in this paper) on the graph provide an approximation of the AMF [black line in Fig. 5 ▸(*a*)]. For all graphs (Figs. 2 ▸, 5 ▸ and 6 ▸), the lines represent the AMFs and the circles represent the correct positions that the atoms in the supercell occupy on the AMF. The initial starting state for the refinement [crosses in Fig. 5 ▸(*a*)] has all atoms on a flat line along **x**_1_ at zero displacement because the average structure is in the same position in each subcell of the initial supercell structure.

**Figure 6:**
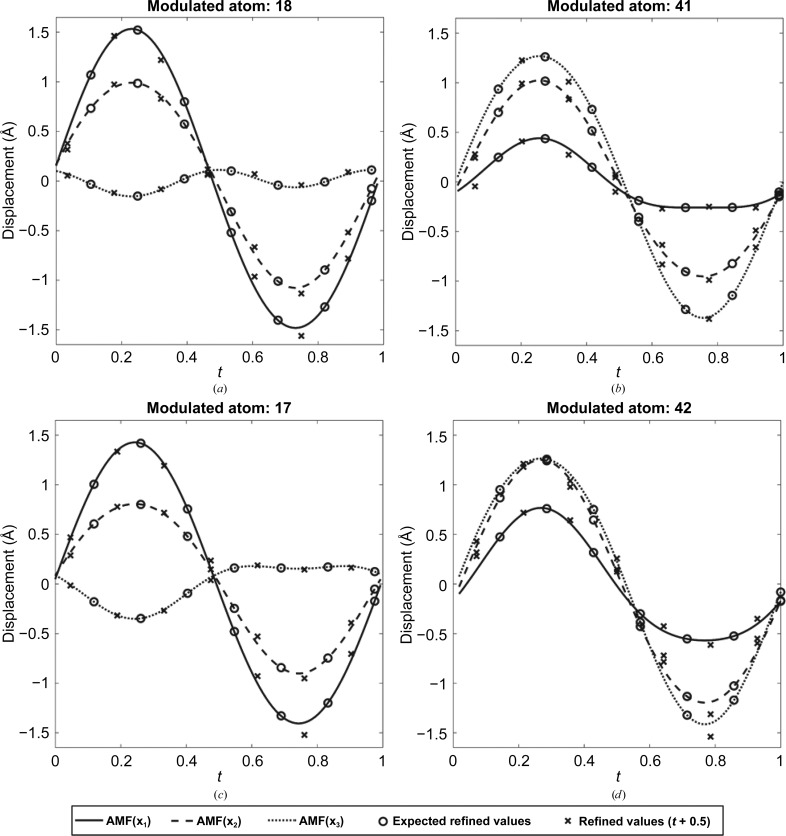
Refinement results for multiple atoms showing all three modulation directions. All atoms refined to the state where *T*_0_ = 1/2. The positions of these atoms on the modulated chain *B* are shown in Fig. 4 ▸. (*a*) Atom 18, (*b*) atom 41, (*c*) atom 17, (*d*) atom 42.

Initially, we reviewed the refinement results by animating the solution with the subcells of the supercell overlaid in superspace order (A, E, B, F, C, G, D; enlarged region of Fig. 2 ▸). In these animations, the displacements for correct refinements show chain *B* rotating back and forth [Fig. 4 ▸(*d*)]. An example can be found in the supporting information (result.gif) which looks the same on comparison with the correct solution (correct.gif in the supporting information). Additionally, the statistics were good, with *R* and *R*_free_ of 2.2% and 2.4%, respectively. Given the observed motion and good statistics, we believed that the refinement was successful. Refinement results [Fig. 5 ▸(*b*)] can also be viewed by a superspace plot of *t* versus displacement where the crosses, in this case, represent the refined positions, and it is clear that they do not line up with the expected positions. The refined solution was shifted half a wavelength in superspace and then plotted [Fig. 5 ▸(*c*)]. From analysis of the shifted plot, it is clear that these seven new states are just a different sampling of the continuum states available along the AMF. When the other two directions are added to the plot [**x**_2_ and **x**_3_; Fig. 6 ▸(*a*)], the case for a phase shift of 0.5 in *t* is made stronger. This same shift is shown plotted for a couple more of the modulated atoms [Figs. 6 ▸(*b*)–6 ▸(*d*)]. For all cases, simply shifting the results by 0.5 in *t* causes the refined values to match the expected AMFs nicely.

### 3.1. Superspace provides an answer   

What happened? If we are just limited to 3D supercell thinking, the result does not make sense; however, if we look at the results within the higher dimensional superspace framework there is a reasonable answer. In this case, the superspace group [19.1 or *P*2_1_2_1_2_1_(0β0)] has two *P*2_1_2_1_2_1_ daughter groups in 3D space. For the first *P*2_1_2_1_2_1_ daughter group the starting phase of the AMFs (*T*_0_) can be selected from one of seven equally spaced positions along *t* where *T*_0_ = *n*/7 and *n* is an integer. The second *P*2_1_2_1_2_1_ daughter group has the starting phase starting at *T*_0_ = *n*/7 + 1/14. For both of these options there are seven choices for the starting value of *n* = 0, 1, …, 6 because of equivalent locations; integer values for *n* > 6 will result in identical positions to *n* = 0, 1, …, 6. For the first daughter group, only one of the choices for *T*_0_ where *n* = 0 results in a 3D cell with no origin shift. For the second daughter group only *n* = 3 which has a *t* offset of 0.5 results in a 3D cell with no origin shift. This second option matches what was observed in refinement.

**Figure 7:**
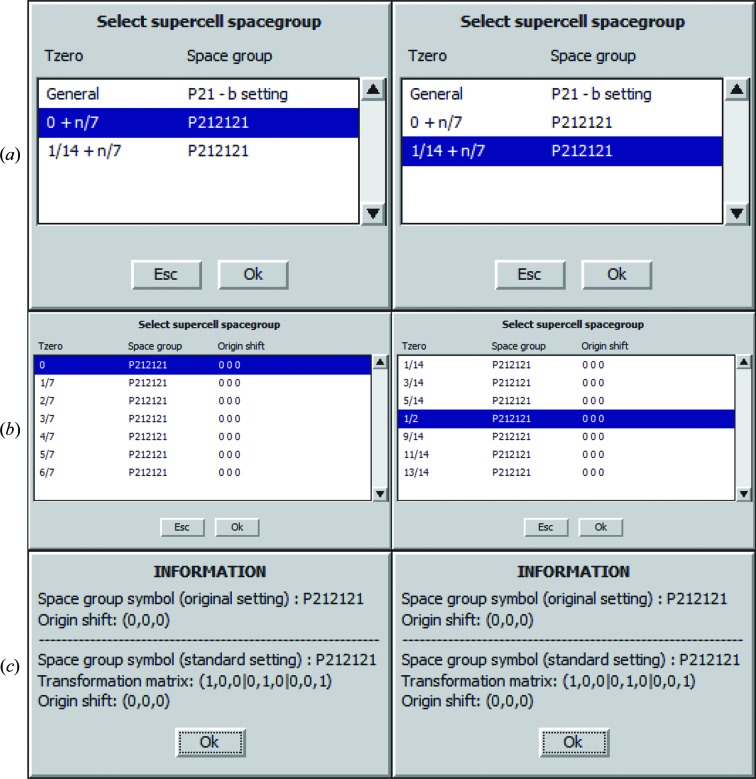
Investigating the available 3D daughter groups for *P*2_1_2_1_2_1_(0β0) in *Jana*2006. (*a*) There are two *P*2_1_2_1_2_1_ 3D daughter space groups: one with *T*_0_ = 0 + *n*/7 and the other with *T*_0_ = 1/14 + *n*/7. *T*_0_ determines the initial state that shows up first in the supercell. (*b*) There are seven options for selecting *T*_0_ for a 7× supercell for each space-group setting. (*c*) Only the highlighted settings in (*b*) for *T*_0_ result in no origin shift. The setting *T*_0_ = 0 is the correct setting and is that used to calculate the ideal structure factors. The setting *T*_0_ = 1/2 is equivalent to the refined result.

The most popular software for refining incommensurate structures of small molecules is *Jana*2006 (Petricek *et al.*, 2006 ▸); unfortunately, there is currently no equivalent package for proteins. It offers a wide range of tools beyond refinement. One of these tools allows the user to explore commensurate approximations (supercells). The daughter 3D cells are derived by *Jana*2006 from the superspace group, and this option can be found under the ‘edit m50’ option in the ‘Cell’ tab (Fig. 7 ▸). *Jana*2006 initially shows the available daughter groups [Fig. 7 ▸(*a*)], then the options for *T*_0_ [Fig. 7 ▸(*b*)] and finally the origin shifts and other changes that may occur to the 3D daughter space group based on the *T*_0_ setting [Fig. 7 ▸(*c*)]. Alternatively, there is an online tool called Superspace Group Finder (https://it.iucr.org/resources/finder/; Orlov *et al.*, 2008 ▸) which can be used to investigate the available 3D daughter groups of a superspace group as well as to work backwards and investigate common superspace groups for a collection of 3D groups.

**Figure 8:**
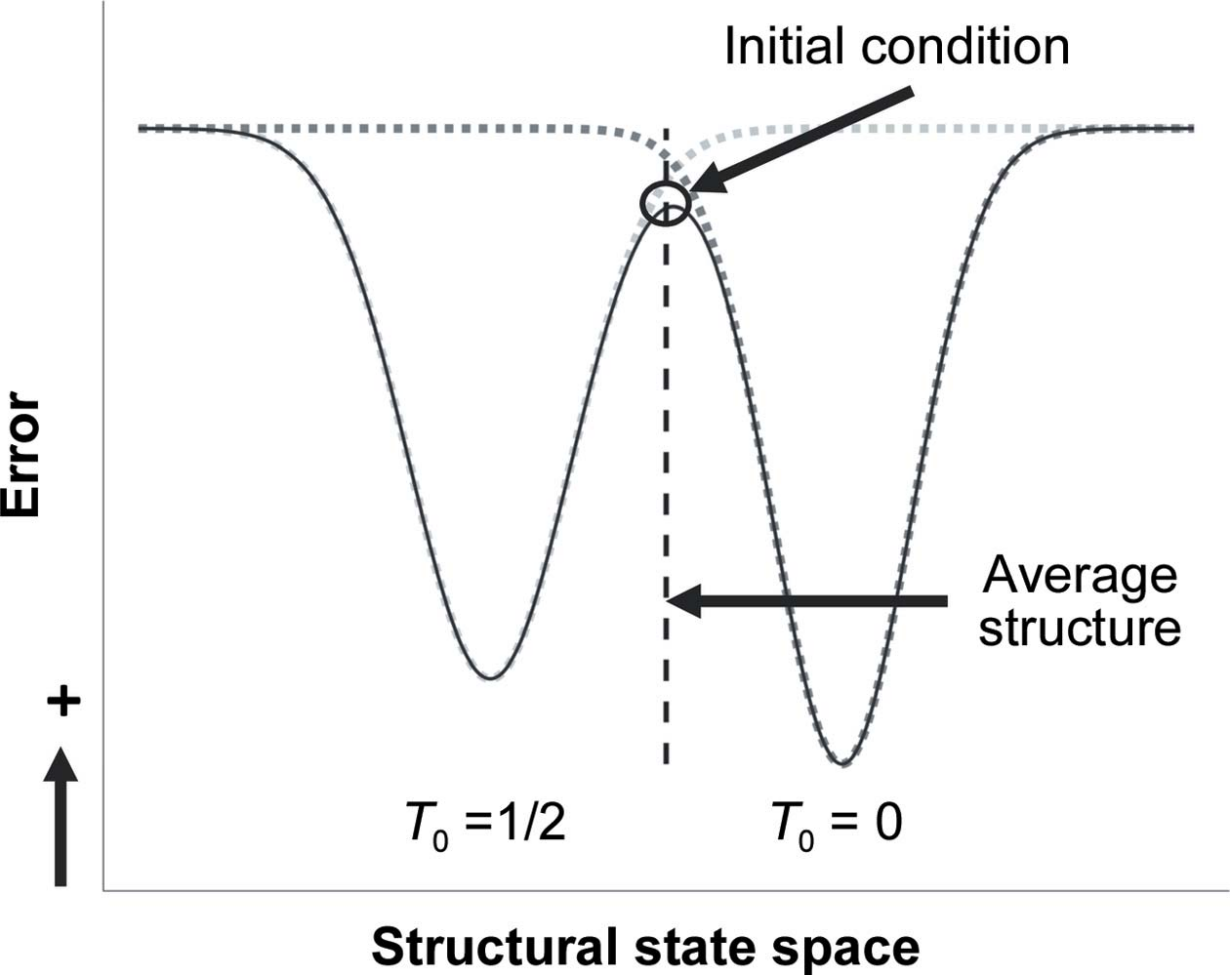
Schematic showing a simplified view of error versus structural states and why the initial condition (black circle), which will be near the average structure (dashed line), tends to result in a refinement where the state is defined by *T*_0_ = 1/2 because the *T*_0_ = 1/2 state will be slightly broader and slightly shallower than the *T*_0_ = 0 state, resulting in a gradient pointing towards the *T*_0_ = 1/2 state. Here, error could be any metric that is used to measure the difference between the observed data and the model.

The next question might be: just how sensitive is the refinement to the starting position in this example? Are there any starting positions that will result in the expected refinement? The sensitivity of the result as a function of the starting position was investigated by pushing the starting position towards one of the two solutions: atoms modulated slightly towards *T*_0_ = 0, the expected solution, or slightly modulated towards *T*_0_ = 1/2, the out-of-phase solution (lines up with the AMF when the *t* positions of the atoms in the refined model are adjusted to *t* + 1/2). Even a small amount of initial movement towards the expected solution (*T*_0_ = 0) will cause the refined solution to converge appropriately (Table 1 ▸). Also, the correct solution does have slightly better statistics. The difference between the two sets, however, is so small that in normal protein refinements (with larger *R* values) these differences might not be interpreted as significant. Although it appears as though the cutoff to converge to the correct solution would be something like better than 0.01% towards the expected solution, this is a rounding limit of the PDB format, where in this case changes to the starting position of 0.01% were indistinguishable from the 0.00% case. It is most likely that in error space the minima describing both structural solution states are equidistant from the initial condition, which would be close to the average position. As the *T*_0_ = 1/2 state results in different reflection intensities, its error well will be both shallower and broader than the correct *T*_0_ = 0 state, and when these states interact in error space there will be a slight tendency toward the *T*_0_ = 1/2 state when starting from near the average position (Fig. 8 ▸). In an effort to verify this model, we plotted initial *R* values (one cycle of refinement) as a function of bias towards one of the two solutions (Supplementary Fig. S1). The graphs demonstrate a very slight tendency towards the *T*_0_ = 1/2 solution around the average position and the *T*_0_ = 0 solution as a global minimum.

For the ToxD case, the modulations were smoothly varying, which makes it easy to detect something strange in the refinement if an atom undergoes a rapid change in position on the *t* plot. In early refinements, there were examples where different parts of the modulated chain converged to different solutions, resulting in some atoms being caught in the middle between these two opposing solutions (data not shown), resulting in noisy, as opposed to smooth, *t* plots of the positions. At the time it was thought that jelly-body refinement corrected this issue, but what happened was that jelly-body refinement forced all of the atoms down one of the two available solutions from superspace and because, as stated earlier, we analyzed the results using only animations, it was not clear that there was an issue. To avoid local minima, *Jana*2006 always performs multiple refinements for supercell approximations by adding small random perturbations to the atomic positions in the hope that this will result in at least one of these refinements finding the global minimum and not just a local minimum. In a commensurate approximation for incommensurately modulated data, the problem is exacerbated. Here, the actual difference in error between different daughter groups will be much smaller and possibly indistinguishable. For an incommensurate case the integration along the entire period of the AMF contributes to reflection intensities, whereas for a commensurate case only a select number of discrete points along the AMF contribute to reflection intensities. The resulting conclusion is that for an incommensurate modulation the superspace (3+1)D description will provide the more accurate picture of what is occurring in the crystal, and this is exactly the conclusion that Wagner & Schönleber (2009 ▸) arrive at after comparing their superspace with their supercell solution.

## 4. Conclusions   

In conclusion, we have revealed that the refined supercell model may not end up in the true atomic positions of the modulated structure owing to the availability of multiple 3D daughter space groups. Using the refined positions of the supercell to fit AMFs should result in approximate AMFs of good enough quality to test whether phase-shifting the atomic positions of the supercell provides a better structural solution. Software tools such as *Jana*2006 or the Superspace Group Finder website can be used to find the appropriate (3+1)D to 3D daughter space group options for testing phase shifts in refinement. For supercell structures, it may be useful to study the atomic positions as plotted in superspace *t* plots to gain more insight into the underlying mechanisms of the displace­ment. Additionally, for supercells, the jelly-body refinement option (or any option like jelly-body refinement in your refinement software of choice) should always be enabled to prevent the model from attempting to refine two solutions simultaneously. In future work, we will employ these methods and observations in the refinement of incommensurately modulated profilin–actin (Lovelace *et al.*, 2008 ▸).

## Supplementary Material

Animated GIF correct.gif: what the correct refinement looks like. DOI: 10.1107/S2059798319011082/rr5176sup1.gif

Animated GIF result.gif: what the refinement looks like. DOI: 10.1107/S2059798319011082/rr5176sup2.gif

Data files. DOI: 10.1107/S2059798319011082/rr5176sup3.zip

Supplementary Figure S1. DOI: 10.1107/S2059798319011082/rr5176sup4.pdf

## Figures and Tables

**Table 1:** Effects of initial conditions on supercell refinement Conditions were biased as a percentage towards a *T*_0_ = 0 or *T*_0_ = 1/2 solution.

Initial condition	Refined solution	*R* (%)	*R*_free_ (%)
100.00% *T*_0_ = 0	*T*_0_ = 0	1.8	1.9
10.00% *T*_0_ = 0	*T*_0_ = 0	1.9	2.0
1.00% *T*_0_ = 0	*T*_0_ = 0	1.9	2.0
0.10% *T*_0_ = 0	*T*_0_ = 0	1.9	2.0
0.01% *T*_0_ = 0	*T*_0_ = 1/2	2.2	2.4
Average structure	*T*_0_ = 1/2	2.2	2.4
0.01% *T*_0_ = 1/2	*T*_0_ = 1/2	2.2	2.4
0.10% *T*_0_ = 1/2	*T*_0_ = 1/2	2.2	2.4
1.00% *T*_0_ = 1/2	*T*_0_ = 1/2	2.2	2.4
10.00% *T*_0_ = 1/2	*T*_0_ = 1/2	2.2	2.4
100.00% *T*_0_ = 1/2	*T*_0_ = 1/2	2.2	2.4
