# Supplementary figures and images for: Supercell refinement: a cautionary tale
Source: Acta Crystallogr D Struct Biol. 2019 Aug 28;75(Pt 9):852–60. doi: 10.1107/S2059798319011082 (PMC6719663; doi:10.1107/S2059798319011082)

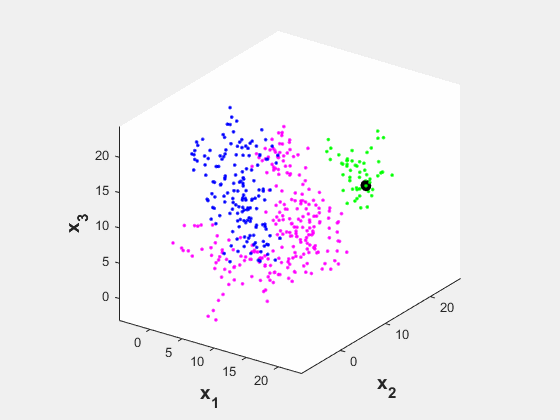

Supplement: Supplementary file 1 [file d-75-00852-sup1.gif]

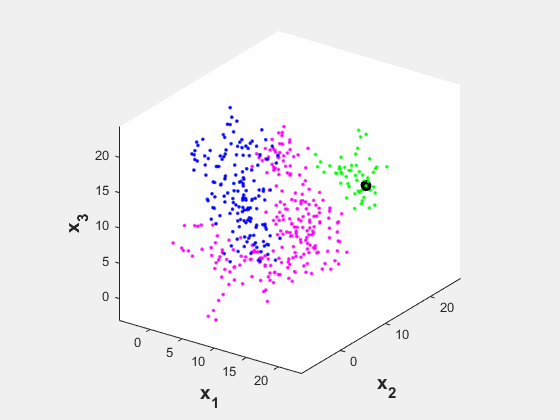

Supplement: Supplementary file 2 [file d-75-00852-sup2.gif]
