# Supplementary material for: Supercell refinement: a cautionary tale
Source: Acta Crystallogr D Struct Biol. 2019 Aug 28;75(Pt 9):852–60. doi: 10.1107/S2059798319011082 (PMC6719663; doi:10.1107/S2059798319011082)
Supplement: Supplementary file 4 [file d-75-00852-sup4.pdf]

STRUCTURAL BIOLOGY

Volume 75 (2019)

Supporting information for article:

Supercell refinement: a cautionary tale

Jeffrey Lovelace, Václav Petrícek, Garib Murshudov and Gloria E. O. Borgstahl

In the paper we argued that the T_0=1/2 solution will have a shallower error well leading to a broader well that when it combines with the error well for the correct solution (deeper and have steeper sides) will cause refinements starting near the average to favor the T_0=1/2 solution.

The error space was approximated by using the initially calculated R values for molecular states that were biased toward one of the two solutions (T_0=0 or T_0=1/2). Figure S1a shows that T_0=1/2 is seems to provide evidence supporting our view about the shape of the error space.

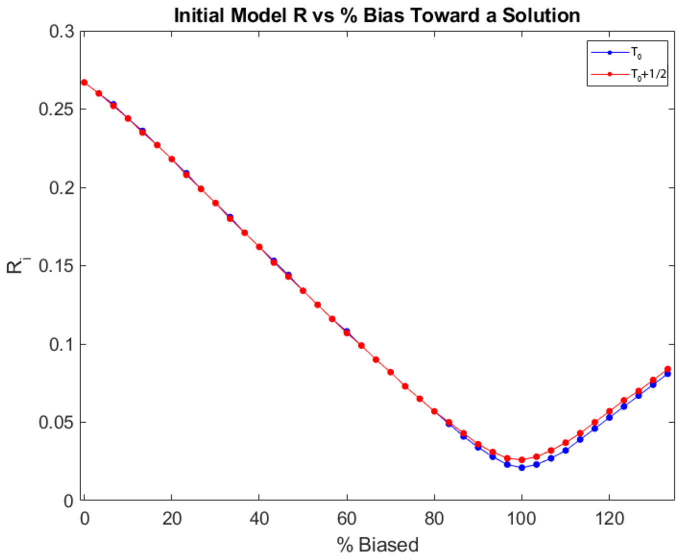

Figure S1(a): Plot of the initial R values as a function of the % bias toward the T_0=0 or the T_0=1/2 solutions. The T_0 solution can achieve a lower R value. A bias of 0% is the average structure 100% represents one of the two solutions and >100% represents an overshooting of the rotation.

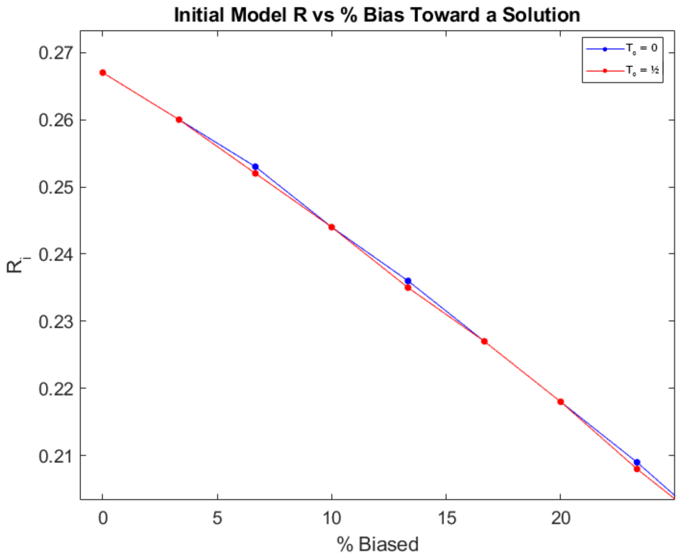

Figure S1(b): Zoomed in region of Fig. S1a near the starting point of refinement. Although T_0=0 has the lowest final R value, T_0=1/2 has (slightly) lower R values for states near the average structure which may cause refinement approaches to favor the T_0=1/2 because of these locally more favorable R values.

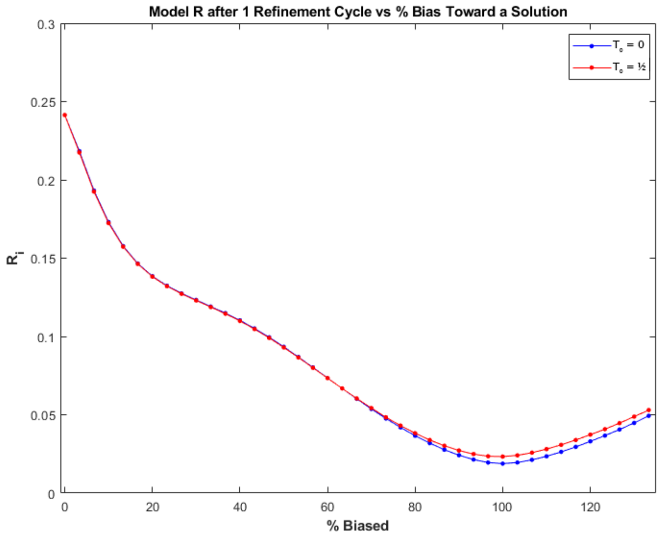

Figure S1(c): Plot of the R values after one cycle of refinement as a function of the % bias toward the T_0=0 or the T_0=1/2 solutions. The T_0=solution can achieve a lower R value. A bias of 0% is the average structure 100% represents one of the two solutions and >100% represents an overshooting of the rotation.

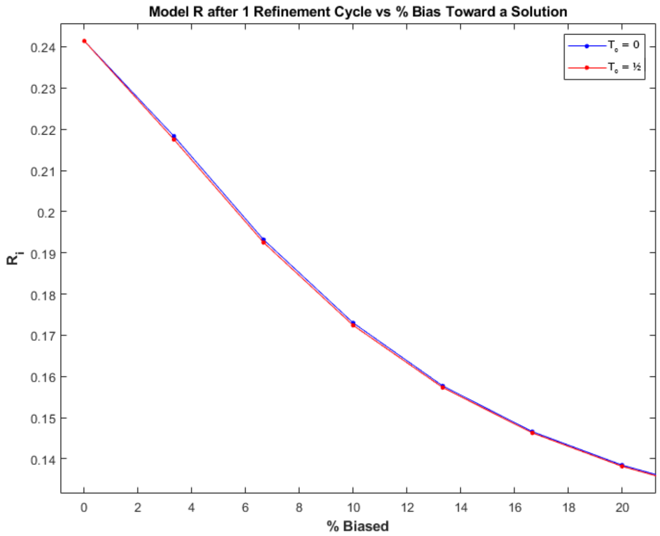

Figure S1(d): Zoomed in region of Fig. S1c near the starting point of refinement. Although T_0=0 has the lowest R value, T_0=1/2 has (slightly) lower R values for states near the average structure which may cause refinement approaches to favor the T_0=1/2 because of these locally more favorable R values.
